# Poncet's Disease: A Case-Based Review

**DOI:** 10.7759/cureus.86101

**Published:** 2025-06-15

**Authors:** Soukaina Zaher, Kawtar Nassar, Saadia Janani

**Affiliations:** 1 Department of Rheumatology, Ibn Rochd University Hospital Center, Faculty of Medicine and Pharmacy of Casablanca, Casablanca, MAR

**Keywords:** antitubercular drugs, polyarthralgia, poncet’s disease, reactive arthritis, tuberculosis

## Abstract

Poncet's disease (PD) is defined by the presence of reactive aseptic oligo or polyarthritis affecting the large joints associated with active pulmonary or extrapulmonary tuberculosis (TB). Its pathogenesis is unknown. It is rare and remains a diagnosis of exclusion. We report a case of PD, clinically manifesting with inflammatory polyarthralgia, that occurred in a 16-year-old patient, and provide a review of the literature.A young 16-year-old Moroccan male was admitted with a history of two-month bilateral symmetrical inflammatory polyarthralgia. It was associated with peripheral lymphadenopathy and hepatosplenomegaly. Laboratory blood tests found an erythrocyte sedimentation rate at 60 mm/h and C-reactive protein at 91 mg/L. Other investigations in search of a possible etiology were normal. Lymph node biopsy showed the presence of an epithelioid cell granuloma with caseating necrosis, suggesting the diagnosis of PD. Under anti-tuberculosis treatment, we noted a significant clinical evolution. Poncet's disease is a rare pathology, not to be forgotten when faced with inflammatory joint symptoms, including inflammatory arthralgia, especially in endemic countries.

## Introduction

Poncet’s disease (PD), first described by Antonin Poncet in 1897, refers to a reactive, aseptic oligo- or polyarthritis associated with active tuberculosis (TB), most often in its extrapulmonary forms. Clinically, it typically presents as symmetrical polyarthritis that may mimic chronic inflammatory rheumatic conditions such as rheumatoid arthritis, although various patterns of joint involvement have been reported [1,2]. The diagnosis of PD is primarily clinical and relies on the exclusion of other causes of arthritis, particularly tuberculous arthritis, autoimmune diseases, and septic arthritis. A key diagnostic feature is the rapid resolution of joint symptoms following the initiation of anti-tuberculosis therapy [3]. Polyarthralgia without frank arthritis, as seen in our case, is an atypical and less frequently described presentation of PD. This can present a diagnostic challenge, especially in TB-endemic settings where distinguishing between true infectious arthritis and immune-mediated manifestations is critical. In this article, we report a case of PD presenting as inflammatory polyarthralgia, and we provide a comprehensive literature review to better characterise the clinical spectrum and diagnostic features of this entity.

## Case presentation

**Figure 1:**
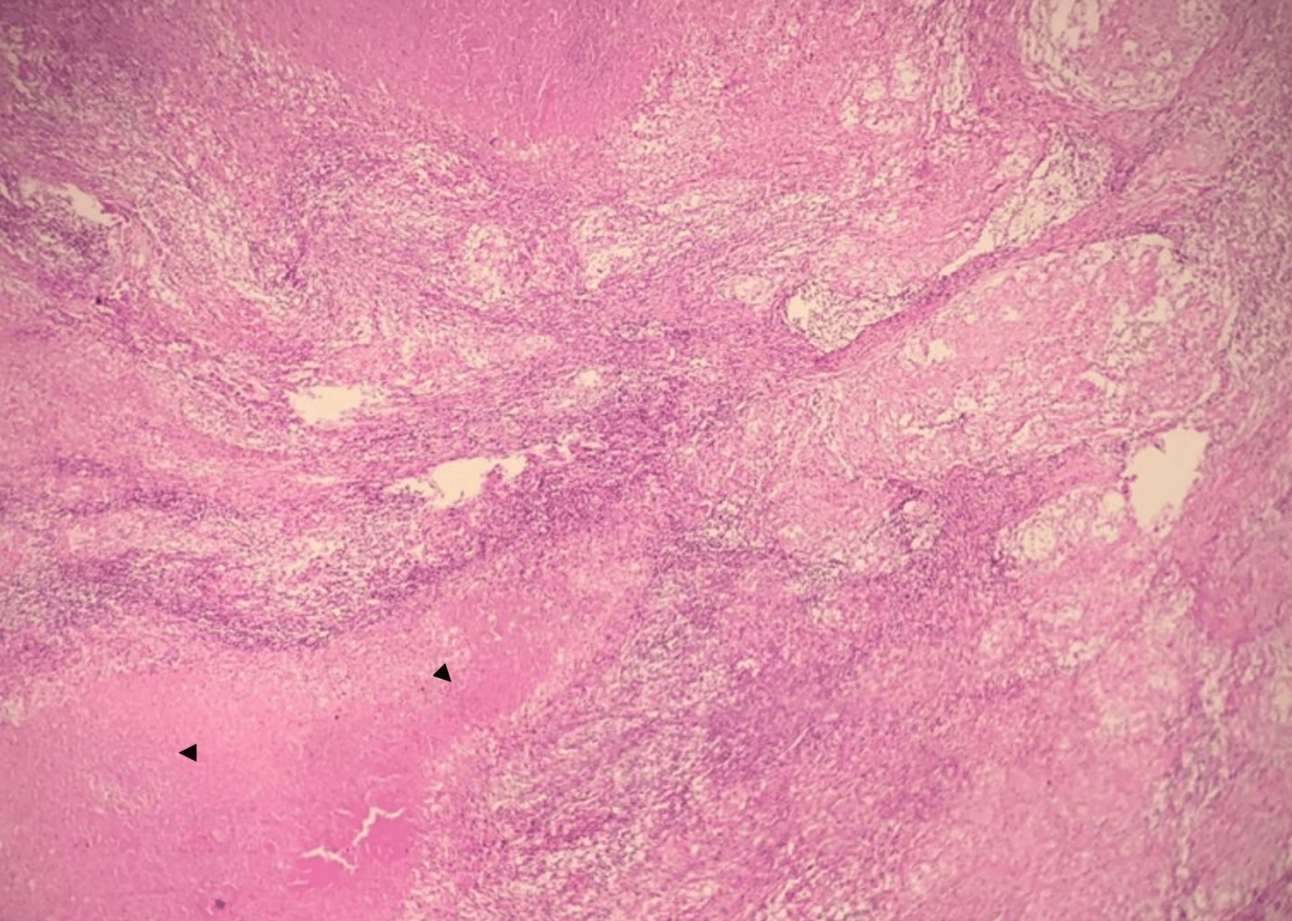
Histological examination of lymph node biopsy with hematoxylin-eosin stain (100X magnification) Black triangles: Caseous necrosis surrounded by granulomatous inflammation.

A 16-year-old Moroccan male was admitted with a history of two-month bilateral symmetrical inflammatory polyarthralgia involving the ankles, knees, hips, and wrists. It was associated with mixed low back pain, inflammatory talalgia, peripheral lymphadenopathy, and hepatosplenomegaly. He had fever and general health deterioration, with asthenia and weight loss. Laboratory blood tests found an erythrocyte sedimentation rate at 60 mm/h and C-reactive protein at 91 mg/L. Other investigations in search of a possible etiology were normal: angiotensin-converting enzyme at 52 UI/L, negative HIV serology, absence of human leukocyte antigen (HLA) B27, negative rheumatoid serology (rheumatoid factor (RF) and Anti-cyclic citrullinated peptides (CCP)) and antinuclear antibodies, as well as the radiological assessment of the involved joints. Cervical lymph node biopsy showed the presence of an epithelioid cell granuloma with caseous necrosis (Figure 1), suggesting a diagnosis of PD, given that the purified protein derivative (PPD) skin test and QuantiFERON-TB gold test were negative.

Note that pulmonary tuberculosis was excluded (Chest X-ray and smear for acid-fast bacilli in morning bronchial secretions were negative). The patient was prescribed anti-tuberculosis drugs according to the national protocol for tuberculosis control: two months of the EHZR combination (Ethambutol -E- / Isoniazid -H- / Pyrazinamide -Z- / rifampin -R-: 275/75/400/150 ) with a weight-dose of three tablets per day, followed by maintenance treatment: four months of the HR combination (75/150) with a weight-dose of three tablets per day. The general health condition improved with a complete resolution of joint symptoms after one week of treatment (Table 1).

**Table 1:** Clinical and biological parameters of our patient as well as the course of the disease SWC: swollen joints count; TJC: Tender joint count; ESR: erythrocyte sedimentation rate; CRP: C-reactive protein; Hb: hemoglobin; MCV: mean corpuscular volume; MCHC: mean corpuscular hemoglobin concentration; WBC: White Blood Cells; AST:  aspartate aminotransferase; ALT: alanine aminotransferase; GGT: gamma glutamyl transferase ; LDH: lactate dehydrogenase.

Parameters	0 Month	One Month	Three Months	Six Months	18 Months	Reference range
SWC	00	0	0	0	0	0
TJC	05	0	0	0	0	0
ESR(mm/1h)	60	42	30	16	10	<15 mm/hr
CRP(mg/l)	91.6	28	16	18	10	<5 mg/L
Hb(g/dl)	10.5	11.9	11.4	10.4	14.3	12.0-13.0 g/dl
MCV(fl)	76.4	80	78	73	85	80-100 fl
MCHC(g/dl)	25.8	31	30	30	32	31-36% Hb/cell
WBC (G/L)	6.8	5.2	6.3	3.9	6.2	4-10 G/L
Platelets (G/L)	585	382	434	392	382	150-400 G/L
AST	25	22	-	-	-	6-25 IU/ml
ALT	23	16	-	-	-	6-25 IU/ml
GGT	15	18	-	-	-	<60 IU/L
Ferritine	12	35	-	-	-	30–400 ng/mL
LDH	345	-	-	-	-	135–225 IU/L

## Discussion

PD is a rare entity, with a slight male predominance [4]. The average age for getting affected is 33.7 years [4]. In Morocco, the largest series reported is by Hammi et al., in which 10 cases were reported [5]. A review of the literature in 2013 found that among the 198 reported cases of PD, 35% were from India, followed by 13.1% from Brazil [4]. Although little is known about PD, advances have been made in recent years. It is well known that tuberculosis is "arthritogenic". Holoshitz et al. had demonstrated an antigenic similarity between a fraction of *Mycobacterium tuberculosis* and human cartilage [6]. Bhattacharya et al. highlighted circulating immune complexes that could be trapped in the synovium of patients with active TB [7]. Southwood et al. found that lymphocyte proliferation assays demonstrated increased purified protein derivative-induced reactivity of synovial fluid lymphocytes compared with peripheral blood lymphocytes [8]. These findings may have key implications in the pathogenesis of PD in a predisposed patient. In those with HLA-DR3 and/or HLA-DR4, there is a T-cell hyper-reactivity to mycobacterial antigens [9]. In addition, several authors have demonstrated the presence of these HLA alleles in patients with PD [10-13]. In fact, Rueda et al. showed that among the 15 cases of PD found in the literature that were tested for HLA-B27, 4 (26.6%) were positive. This is notable considering the general population prevalence of HLA-B27 positivity in the countries included in the study ranges from 0.3% to 14%.

PD presents as a non-destructive aseptic arthritis developing in the presence of active TB [14]. It mainly affects large joints without axial involvement. The most frequently affected joints are: ankles 63.3%, knees 58.8%, wrists 29.1%, and elbows 23.1% [2,14]. Out of the total, 40% of patients present with oligoarthritis, 27.6% with polyarthritis, and 24.6% with mono arthritis. Indeed, inflammatory arthralgia can also be a clinical form of PD, as in the case of our patient. No extra-articular manifestation was found, and only a few patients presented with erythema nodosum [4]. In order to better characterise the clinical spectrum of Poncet’s disease, we reviewed and summarised a selection of well-documented cases reported in the literature across various regions and age groups. Table 2 presents an overview of these cases, including patient demographics, clinical presentation, diagnostic approach, treatment modalities, and outcomes. This summary highlights the variability of PD presentations, from monoarthritis to frank polyarthritis, affecting both adults and children. It also illustrates the importance of excluding differential diagnoses such as septic arthritis and autoimmune diseases through histological, microbiological, and immunological workups. Importantly, several cases underline that PD may manifest after the initiation of anti-TB therapy, possibly due to immune reconstitution or paradoxical response, reinforcing the immunologically mediated pathogenesis of this entity [1,2,4,5,15-20].

**Table 2:** Summarizes representative cases of Poncet’s disease reported in the literature, highlighting their clinical presentations, diagnostic approaches, treatments, and outcomes. * Case Report and Literature Review; ** Mean age of patients reported in the literature review; *** Mean age of patients reported F: Female; M: Male; RA: Rheumatoid arthritis; TB: Tuberculosis; Anti-TT: Anti-tuberculosis treatment; NSAIDs: Non-Steroidal Anti-Inflammatory Drugs; H: Isoniazid; R: Rifampicin; Z: Pyrazinamide; E: Ethambutol.

Author (Year)	Number of cases	Age (years) /Sex	Clinical presentation	Diagnosis method	Treatement	Outcome
Rueda et al. (2013) *	1 198	36/M 33.7**(2-78) 78F 84M	Polyarthritis (elbows, knees, ankles), myalgias, fever. Non-destructive oligoarthritis/polyarthritis	Open lung biopsy (caseating necrosis), exclusion of RA & other infections. Active TB (43%), positive Mantoux test, sterile synovial fluid, exclusion of RA and other infections.	Anti-TT	Resolution in 7 days. Favorable response in less than 2 months in the majority of cases
Sasaki et al. (2015)	01	50 / F	RA-like polyarthritis	Biopsy (caseating granuloma)	Anti-TT + corticosteroids	Resolution in 1 month
Arora et al. (2016)	01	25 / M	Polyarthritis (ankles, knees, wrists)	Clinical + Radiological	Anti-TT + NSAIDs	Resolution in 2 weeks
Hammi et al. (2016)	10	34/F++	Polyarthritis	NA	Anti-TT + NSAIDs	Favorable response
Sharma et al. (2016)	23	33***(14-60) 12F 11M	Oligoarthritis (13), Polyarthritis (10)	Confirmed extra-articular TB, positive Mantoux test, non-destructive arthritis, exclusion of other causes	Anti-TT	Complete resolution within 4 to 8 weeks
Adhi et al. (2017)	02	25/F 45/M	Polyarthritis	Confirmed TB, exclusion of other diagnoses	Anti-TT Anti-TT+ corticosteroids	Complete remission within days; no recurrence after 3-year follow-up Rapid improvement within 10 days; full recovery confirmed at 5-month post-treatment follow-up
Verma et al. (2021)	01	07/M	Polyarthritis, alopecia, splenomegaly	Mantoux+, radiography, synovial fluid negative, Sharma & Pinto criteria	ATT Category I (2HRZE/4HR)	Complete improvement
Higashiguchi et al. (2022)	01	82/M	Polyarthritis (fingers, wrists, ankles) after 6 weeks ATT	Imaging, synovial biopsy (non-specific inflammation), exclusion of TB & RA	ATT + corticosteroids (Prednisolone 15 → 5 mg/day)	Pain resolution, no joint damage at 6 months
Mohamedali et al. (2023)	01	38 / F	Polyarthritis (knees, wrists, MCP joints)	GeneXpert+, chest X-ray, exclusion of autoimmune diseases	2HRZE/4HR	Complete resolution, normal labs at 6 months
Zengin et al. (2024)	01	25/F	Polyarthritis	Laparoscopy/histology (not specified), exclusion of other causes	Quadruple anti-TT	Complete resolution of arthritis and adnexal mass

The diagnosis of PD in most cases is clinical. Sharma et al. proposed criteria in which non-erosive and non-deforming arthritis is a key diagnostic element [15]. The simultaneous diagnosis of extra-articular TB and the complete response to antituberculosis therapy without sequelae were the most discriminating clinical characteristics that differentiated PD from other inflammatory arthritis. Therefore, these criteria have been defined as major criteria. Other signs of PD, such as the positivity of the intradermal reaction to tuberculin, the presence of other phenomena of hypersensitivity (such as tuberculids), and the absence of axial involvement, were considered as minor criteria. Patients are classified as probable or possible cases initially, and then upgraded to a definite PD after a good response to the antituberculosis therapy [15]. In our case, the diagnosis of PD was retained according to these criteria.

Treatment is based on anti-TB drugs, which relieve symptoms over a variable period of time, ranging from five days to four weeks, with some cases requiring the use of non-steroidal anti-inflammatory drugs and corticosteroids [5]. For our patient, the relief was considered complete after one week of antituberculosis therapy administration according to the national protocol for tuberculosis control. PD has a favorable prognosis. No case of chronic arthritis has been reported to date. Our patient was progressing well on antituberculosis therapy without joint complications after a two-year follow-up.

## Conclusions

Inflammatory polyarthralgia is also a clinical presentation of Poncet's disease alongside oligoarthritis and polyarthritis. PD should be considered in the differential diagnosis of patients with prolonged fever with joint symptoms and active tuberculosis infection, especially in high-prevalence areas. The prognosis is good with complete resolution of symptoms under anti-tuberculosis treatment, without residual joint damage as in the case of our patient. PD should not be overlooked in the diagnostic hypotheses of inflammatory rheumatism because the usual treatment of inflammatory arthritis based on immunosuppression can lead to the spread of the infection. In light of its diagnostic challenges and therapeutic implications, the development of standardized diagnostic criteria and the creation of multicentre registries would greatly enhance the recognition and characterization of Poncet’s disease.
